# De Witt County Medical Society

**Published:** 1860-05

**Authors:** C. Goodbrake

**Affiliations:** Sec’y.


### DE WITT COUNTY MEDICAL SOCIETY.

The Society met in annual session, at the office of Dr. Adams, at Clinton, on Tuesday, the 3d. day of April, 1860, at 10 o'clock, A. M. Present, Drs. B. S. Lewis, W. W. Adams, T. K. Edmiston, Z. H. Madden, J. H. Tyler, John Wright, Evan Richards, and C. Goodbrake, Also Dr. Davis, of Wapello, and Mr. Edwin Gideon, medical student.

The President, Dr. Adams in the Chair.

The secretary being absent, Dr. Goodbrake was appointed Secretary pro tem.

The minutes of the last meeting were read and approved.

The following officers were elected for the ensuing year:

*President*—Dr. J. H. Tyler.

*Vice President*—Dr. Evan Richards.

*Treasurer*—Dr. Z. H. Madden.

*Secretary*—Dr. C. Goodbrake.

*Censors*—Drs. W. W. Adams, T. K. Edmiston, and John McHugh.

*Delegates to the Illinois State Medical Society*—Dr. T. K. Edmiston, Dr. John McHugh and Dr. Z. H. Madden.

*Delegates to the American Medical Association*—Dr. John Wright and Dr. Joseph C. Ross.

Dr. Tyler, on taking the Chair, delivered a most excellent address, which was received with marked attention.

Dr. Lewis, the retiring President, delivered a well written valedictory address. Subject—the "Ingratitude of the public to the Medical Profession."

On motion of Dr. Goodbrake, the thanks of the Society were tendered to Dr. Lewis for his excellent address, and a copy requested for publication in the *Central Transcript*.

Dr. Richards reported a very peculiar case of injury of the chest—the patient having been thrown from a sulky.

Dr. Wright read the report of a case of typhoid fever, with the post mortem appearances. Accompanying this report, the doctor exhibited a portion of ileum which was perforated in four different places. A very spirited discussion arose on the subject of typhoid fever, in which most of the gentlemen present partook.

On motion, Typhoid fever was made the subject for discussion at the next meeting.

The President appointed Drs. Adams and Edmiston, to read essays at the next meeting.

On motion of Dr. Richards, Dr. S. W. Noble, of Le Roy, was appointed to write an essay on Puerperal Peretonitis.

On motion of Dr. Lewis, the thanks of the society were tendered to Dr. Edmiston and lady, for the excellent dinner furnished the members, at the Doctor's residence.

On motion, the Secretary was ordered to furnish a copy of these proceedings to the Editors of the *Chicago Medical Journal*, for publication.

On motion, the Society adjourned, to meet in quarterly session, at Santa Anna, on the first Tuesday of July next, at 10 o'clock A. M.

C. GOODBRAKE, Sec'y.

